# Obesity and Postoperative Cognitive Dysfunction: A Curious Association

**DOI:** 10.7759/cureus.42436

**Published:** 2023-07-25

**Authors:** Camden I Burns, Anto Boghokian, Varun Soti

**Affiliations:** 1 Anesthesiology, Lake Erie College of Osteopathic Medicine, Elmira, USA; 2 Cardiology, Lake Erie College of Osteopathic Medicine, Elmira, USA; 3 Pharmacology and Therapeutics, Lake Erie College of Osteopathic Medicine, Elmira, USA

**Keywords:** cognitive dysfunction, general surgery, perioperative outcomes, neurocognitive deficit, obesity, postoperative cognitive dysfunction

## Abstract

Postoperative cognitive dysfunction (POCD) is a medical condition that impacts cognitive function after surgery, particularly major procedures. Patients with POCD may experience physical symptoms, such as depression, anxiety, and fatigue, severely undermining their quality of life. Research establishes the connection between obesity and cognitive dysfunction since patients diagnosed with obesity are more susceptible to cognitive decline. Although obesity poses a significant risk factor for cognitive impairment, the link between obesity and POCD is still inadequately understood. Therefore, this systematic review explores the correlation between obesity and POCD by detailing potential mechanisms underlying this relationship and identifying areas for further research. Following the guidelines for systematic reviews, we conducted a literature search between August 2022 and April 2023, which identified studies with a substantial number of patients with POCD after major surgeries, including coronary artery bypass grafting, gastrointestinal procedures, cholecystectomy, and carotid endarterectomy. Our findings also demonstrated that a significant percentage of these had obesity, which was statistically significant as a risk factor for cognitive decline. Pathological processes, such as changes in vascular endothelium integrity, systemic inflammation induced by obesity, and apolipoprotein E-epsilon-4 expression, have been identified to contribute to POCD after surgery. Despite the promising results, there remains a gap in the literature. Thus, it is crucial to investigate the relationship between obesity and POCD further, uncover more potential underlying pathophysiological processes, and identify therapeutic targets. These measures would enable healthcare practitioners to prevent or reduce cognitive dysfunction associated with obesity in surgical patients.

## Introduction and background

Postoperative cognitive dysfunction (POCD) is a medical condition characterized by decreased cognitive functioning following surgery, especially major surgical procedures. Specifically, it implies changes or loss of preoperative intellectual abilities, such as memory, learning, orientation, concentration, and decision-making in post-surgical patients. However, the definitions of POCD have varied among research groups, attributing to differences in specific neuropsychological or neurocognitive tests, cognitive decline calculation methods, and reported cognitive decline timing and magnitude. For example, some researchers have defined POCD as cognitive decline reported between 17 and 43 days after undergoing a major surgical procedure or a complicated postsurgical recovery. Whereas others have noted it as cognitive impairment reported after 30 days and persisting for a year or longer after a major surgical procedure [1]. Unfortunately, patients diagnosed with POCD may experience depression, anxiety, fatigue, and other physical symptoms, significantly reducing their quality of life [2].

The surgical procedure itself is believed to trigger a systemic inflammatory response, serving as a significant factor in POCD development. Proposed mechanisms involve pre-existing, yet undetected, neuroinflammation or neurodegeneration, which makes the brain more susceptible to acute systemic inflammatory response. Consequently, this leads to accelerated neuroinflammation and neurodegeneration, ultimately resulting in neurocognitive impairment. However, little is known about the potential impact of patient-specific and perioperative factors on the progression of POCD caused by initial inflammatory responses to surgery [1].

Notably, there is a connection between obesity and cognitive dysfunction, with research indicating that obese individuals, estimated to be more than 650 million worldwide [3], are more likely to have cognitive decline than non-obese individuals [4]. However, the relationship between obesity and POCD has not been thoroughly researched. Obesity poses a significant risk for numerous diseases, including type 2 diabetes, hypertension, and other cardiovascular diseases [5]. In addition, it has been linked to inflammation [6]. There is a possibility that patients who are obese or carry risk factors for obesity are at a greater risk of developing POCD after undergoing significant surgeries [7].

Therefore, this systematic review explores the relationship between obesity and POCD. It also aims to analyze potential mechanisms that underlie the connection between these two conditions while identifying areas for future research. Our review provides insights into possible cognitive decline in patients, specifically those who are obese, after major surgeries. It may help healthcare providers to develop strategies to manage cognitive function in such patient cohorts perioperatively.

## Review

Literature search and study selection

**Figure 1:**
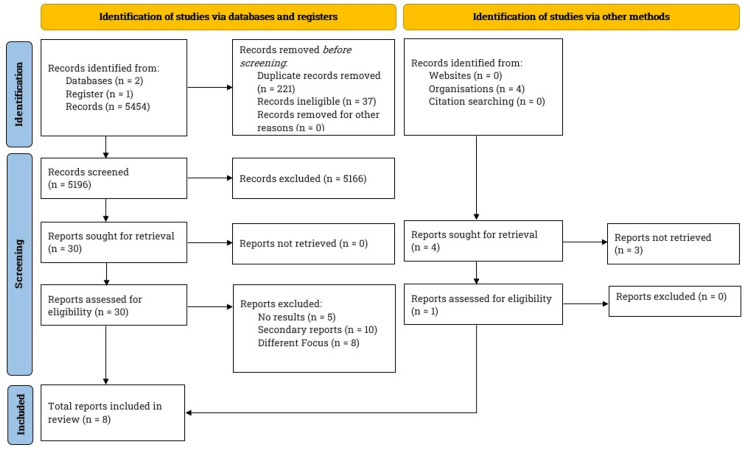
Literature search and study selection. We searched PubMed, BioMed Central, and ClinicalTrials.gov for this review. Our inclusion criteria specified articles written in English and complete clinical studies focusing on the underlying postoperative cognitive dysfunction, obesity, and mechanisms underlying the two conditions. After screening the studies, we narrowed the number down to eight.

Between August 2022 and April 2023, we conducted a comprehensive literature search following the Preferred Reporting Items for Systematic Reviews and Meta-Analyses (PRISMA) guidelines [8]. Our search included notable databases: PubMed, BioMed Central, and ClinicalTrials.gov. Figure 1 depicts the PRISMA flowchart, which outlines the study selection and the total number of studies reviewed for this article.

We implemented inclusion and exclusion criteria (refer to Table 1). We also limited our search to studies published between 2001 and 2023, excluding preliminary clinical trials with partial or no results and studies not published in English. We included relevant studies and assigned a level of clinical evidence based on previous literature [9].

**Table 1:** Study selection criteria. Studies published in English between 2001 and 2023, focusing on postoperative cognitive dysfunction in obese patients and meeting the inclusion criteria were included in the review.

Inclusion criteria	Exclusion criteria
Prospective clinical studies (randomized and non-randomized clinical trials)	Preclinical studies
Double-blinded controlled studies	Case series and case reports
Single-blinded controlled studies	Meta-analysis
Longitudinal studies	Systematic and narrative reviews
Observational studies	Opinions and commentaries

The search terms included “Postoperative Cognitive Decline,” “Postoperative Cognitive Decline AND Obesity,” “Postoperative Cognitive Decline AND Body Mass Index,” and “Postoperative Cognitive Decline AND Body Weight,” “Postoperative Cognitive Decline AND Obesity AND Neuroinflammation,” “Postoperative Cognitive Decline AND Obesity AND Systemic Inflammation,” “Postoperative Cognitive Decline AND Obesity AND Vascular Endothelium,” and “Postoperative Cognitive Decline AND Obesity AND Blood-Brain Barrier.”

Association between obesity and POCD

In a prospective longitudinal trial, Newman et al. (2001) analyzed 261 patients undergoing elective coronary artery bypass grafting (CABG) by conducting neurocognitive assessments before surgery, at discharge, and six weeks, six months, and five years later. Their findings showed a significant decline in cognitive function across all domains. About 53%, 36%, and 24% of patients demonstrated cognitive deterioration at discharge, six weeks, and six months, with 42% at five years (p < 0.001) after surgery. These patients had a significant medical history of diabetes, hypertension, previous myocardial infarctions, and angina. Although obesity is related to these critical risk factors, the study did not report whether patients were overweight or obese. Despite this, the research findings suggested that POCD was present in patients with obesity risk factors, including diabetes and hypertension, for up to five years following CABG surgery [10].

A few years later, Kadoi and Goto (2006) sought to identify the risk factors for POCD after CABG, recruiting 88 patients scheduled for elective CABG. They administered neurological and neuropsychological tests to study patients one day before and six months after the procedure. Interestingly, 54% had a remarkable medical history of hypertension, and 36% were significant cigarette smokers. Statistical analysis of the study findings showed that at six months, the incidence rate of cognitive dysfunction was 27.3%, with a substantial relationship between the development of POCD, renal failure (p < 0.001), and diabetes mellitus (p < 0.001). However, the researchers failed to record the body mass indices (BMI) of the study patients, which could have given the overweight or obese status of the patients. It is worth noting that hypertension and diabetes, along with smoking, are significant risk factors for obesity. Nonetheless, the study revealed remarkable results that risk factors for obesity, such as diabetes and hypertension, were strongly associated with POCD in the study patients. Based on their findings, the researchers could conduct a clinical trial with a larger sample size and more follow-ups to investigate the direct association between obesity and POCD while elucidating the underlying mechanisms [11].

In a groundbreaking clinical trial, Heyer et al. (2005) examined cognitive dysfunction in obese and non-obese patients after uncomplicated carotid endarterectomy (CEA). Specifically, the study analyzed whether cognitive dysfunction was related to the presence of the apolipoprotein E (APOE) epsilon 4 (ε4) allele, associated with worse outcomes following stroke. The authors enrolled 75 patients and evaluated them before and one month after surgery using a standard battery of neuropsychological tests to assess neurocognitive deficits. They also genotyped all patients for APOE by the restriction fragment length polymorphism analysis and used logistic regression to analyze APOE-ε4 and obesity, along with other risk factors. The results showed that APOE-ε4-positive and obese patients were more likely to experience a neurocognitive injury post-CEA. Specifically, 42% of APOE-ε4-positive patients and 33.3% of obese patients experienced neurocognitive dysfunction compared to 5% of APOE-ε4-negative patients (p < 0.001) and 6.3% of non-obese patients (p < 0.03), respectively. It was the first study to demonstrate a direct association between POCD and obesity and to suggest that APOE-ε4-allele in obese patients may be a cognitive deterioration mechanism. Despite these remarkable findings, the notable lack of follow-up of patients beyond one-month post-CEA was a significant limitation. Therefore, a more extensive study with long-term follow-up is warranted to augment these results further and elucidate the other underlying mechanisms that might explain the development of POCD in obese patients with the APOE-ε4 allele [12].

Zhu et al. (2014) aimed to check if a blood transfusion in patients after hip replacement surgery induced POCD. Interestingly, they unexpectedly found that body weight could predict POCD. Their prospective study involved 205 patients, and they evaluated the patients before and seven days after surgery for neurocognitive function. Among the patients who required blood transfusion, 56 developed POCD at a seven-day follow-up, and older age, lower educational level, and blood transfusion involving more than three units were independent risk factors for POCD. The researchers did not track BMI, as it was not their primary focus but recorded body weights at baseline. They found that patients with neurocognitive decline seven days after surgery had a body weight of 61.1 ± 11 kilograms (kg) at baseline compared to their counterparts. The study findings indicated that higher body weight could influence POCD, highlighting a direction for further investigation to identify mechanistic targets, leading to enhanced patient care [13].

Pérez-Belmonte et al. (2015) assessed POCD after an off-pump CABG and its associated risk factors in a research study. The prospective study enrolled 36 patients undergoing off-pump CABG, performing neuropsychological test battery one month before surgery and three follow-up tests at one, six, and 12 months after surgery. Results showed that more than 50% of patients had maximum postoperative cognitive impairment six months after the surgery (p < 0.01). More than 30% of patients still suffered from it at 12 months (p < 0.01). Interestingly, the study found obesity as one of the risk factors for POCD in patients with a 39% rate (p < 0.05). The study also identified smoking, diabetes mellitus, and diastolic blood pressure among the many other risk factors for POCD [14].

Pérez-Belmonte et al. (2015) corroborated Heyer et al.'s (2005) findings [12], providing another piece of evidence on the link between obesity and POCD. This evidence can be helpful in counseling patients before surgery, emphasizing the significance of postoperative neurocognitive evaluation and necessary neurological intervention or rehabilitation. Also, future investigations can target obesity and POCD from a mechanism and treatment standpoint [14].

In their recent study, Heyer et al. (2015) investigated the potential link between statin use and POCD in patients undergoing CEA. The study also assessed the impact of factors such as obesity on cognitive outcomes. It administered neuropsychological tests to 585 patients before and within 24 hours after surgery. The findings revealed that 145 patients had significant cognitive impairment post-surgery, and 127 of those with cognitive impairment had a body mass index above 30 kg per meter square (m^2^). These results are consistent with previous research findings by Heyer et al. (2005) [12] and Pérez-Belmonte et al. (2015) [14], further establishing the relationship between obesity and POCD. However, the study had limitations, including the lack of multiple neurocognitive assessments at various time points post-surgery. Additionally, the study focused solely on long-term patient survival rates with and without statin use rather than evaluating the long-term impact of risk factors such as obesity on the neurocognitive decline after CEA. Nevertheless, these findings provide a foundation for future investigations of obesity's relationship with POCD. Such research could help identify critical therapeutic targets and improve postoperative patient care [15].

He et al. (2019) also conducted a study that found a link between higher baseline body weights and POCD in patients undergoing gastrointestinal surgery. In this prospective observational study, the researchers recruited 124 patients aged 60 years and above scheduled for the procedure and administered them neurocognitive tests before and seven days after surgery. Interestingly, those who suffered from POCD, evaluated seven days after the surgery, had a body weight of 60.9 ± 9.8 kg than 57.4 ± 10.2 kg body weight of those who did not suffer from POCD. Even though the difference in body weight was not statistically significant (p = 0.06), it supports the hypothesis that obesity is linked to POCD, as corroborated by previous studies. However, studies with larger sample sizes can further strengthen these results statistically [16].

Potential mechanisms underlying obesity and POCD

As presented above, the clinical evidence establishes a relationship between obesity and POCD, but what lies beneath? There can be a variety of pathologies contributing to POCD in obese patients after surgeries. For instance, systemic inflammation, vascular pathologies, blood-brain barrier (BBB) breaches, and neuroinflammation may be underlying factors. Alla (2021) delved into these factors by examining grade I and II obese patients. These patients had a BMI of 30-34.9 kg/m^2^ and 35-39.9 kg/m^2^, respectively, and had been obese for at least 10 years. The study aimed to investigate how obesity relates to POCD by assessing whether exogenous endothelioprotectors and anti-inflammatory agents would affect cognitive function in grade I and II obese patients. The study recruited 84 obese patients with comorbidities and divided them into three groups. Group I (serving as a control) received the standard protocol for perioperative intensive care. Group II received a meglumine sodium succinate intravenously (IV) with the standard preoperative protocol before surgery. Finally, Group III also adhered to the standard preoperative protocol but received L-arginine hydrochloride IV before the surgery. Meglumine sodium succinate, an anticoagulant, protects against lipid peroxidation and reactive oxygen species. On the other hand, L-arginine hydrochloride, an endothelial protector, protects against vascular damage [17].

Comparing their results five days after surgery, Groups II and III showed higher cognitive test scores than Group I. Group II scored 26.9 ± 0.2, and Group III scored an impressive 29.6 ± 0.4, outscoring Group I's 26.1 ± 0.2. The researchers also noted that the five-word test score was substantially higher for Groups II and III than for Group I, at 8.6 ± 0.1 and 9.7 ± 0.2, respectively, as opposed to 8.2 ± 0.4, Group I's score. Also, the addition of protective factors to the blood reduced systemic inflammation. Specifically, von Willebrand Factor and antigen ratios were significantly lower in Groups II and III patients five days after surgery [17].

The study's results highlight that safeguarding the vascular endothelium is crucial in reducing inflammation and enhancing cognitive performance following cognitive assessment tests. Future research should expand the sample size and examine the patients' comorbidities to strengthen these findings. Additionally, a fourth group to test the prophylactic administration of a direct anti-inflammatory drug like intravenous ketorolac would provide a helpful comparison [17].

Interestingly, Alla (2021) proposed that vascular endothelial dysfunction and inflammation underlie the occurrence of POCD in obese patients following surgery [17]. Therefore, taking measures to preserve the integrity of the vascular endothelium and reduce inflammation is vital to maintain and improve cognitive function. It is important to note that a breach of the BBB could lead to cognitive deficits and neuroinflammation, ultimately culminating in POCD in obese patients. Studies exploring BBB compromise in obese patients following surgery could provide insight into the exact mechanism of POCD [18].

Moreover, research has shown that certain factors like inflammatory markers, including C-reactive protein, complement C3, and interleukin-6, positively correlated with increased adiposity, a risk factor of cerebrovascular disease, and decreased cortical thickness, ultimately leading to cognitive deficits in obese patients. However, research endeavors regarding these factors in obese patients who suffer from POCD are lacking. Such investigations will be crucial to identify the underlying pathophysiological process that results in POCD in obese patients [19].

Neuroinflammation affects the plasticity of neurons, leading to cognitive impairment in obese patients. Samara et al. (2020) demonstrated that neuroinflammation plays a role in the restructuring of neuronal plasticity in significant cognitive regions of the brain. The researchers used diffusion-based spectrum imaging (DBSI) to evaluate the presence of neuroinflammation and provide insight into the microstructural integrity of the white matter in obesity. They hypothesized that obese individuals, compared to non-obese individuals, would exhibit a higher DBSI-restricted fraction (RF) (indicating greater cellularity related to neuroinflammation), a higher DBSI-hindered fraction (HF) (indicating increased edema), and a lower DBSI-derived fiber fraction (FF) (indicating reduced axonal density). They investigated a cohort of both obese and non-obese individuals (cohort 1), as well as a more diverse convenience sample (cohort 2), to confirm the presence of similar patterns related to BMI status. Cohort 1 consisted of 25 obese and 21 non-obese participants and had a more significant proportion of African Americans than non-obese participants. Cohort 2 had 18 obese and 41 non-obese participants [20].

The research team conducted magnetic resonance imaging on a Siemens Trio 3T scanner (Siemens Healthineers, Erlangen, Germany) with a 20-channel head coil for both groups, then applied tract-based spatial statistics for whole-brain white matter analyses. Then, they compared the isotropic and anisotropic diffusion measures derived from DBSI between the obese and non-obese groups. A significant number of white matter tracts showed that the obese group had significantly higher DBSI-RF and lower DBSI-FF, with the DBSI-RF and DBSI-FF values in the hippocampus being significantly higher and lower, respectively. These findings indicate that neuroinflammation is remarkably present in the white matter tracts and hippocampus of obese individuals in both cohorts (cohort 1: p = 0.045; cohort 2: p = 0.008). It is essential to note that the researchers' work incorporated rigorous methodology, providing validity and generalizability to the results [20].

Though these significant findings demonstrate alterations in the structural integrity of the brain leading to neuroinflammation in obese patients, there has been no research to identify structural abnormalities in the brain's white matter and hippocampus leading to POCD after surgery in obese patients. Therefore, researchers must explore this possibility further. It will be fascinating to understand more about the relationship between obesity and POCD and how alterations in the brain's structural integrity caused by obesity may lead to POCD after surgery [20].

Another study conducted by Kullmann et al. (2020) further established the link between obesity and inflammation in the brain. They utilized quantitative magnetic resonance imaging (qMRI) to quantify accurate pathophysiological processes in the brain, for example, using proton density imaging to evaluate the brain water content, which alters in various pathologies, including inflammation. The researchers recruited 115 normal-weight, overweight, and obese patients (BMI range: 20.1-39.7 kg/m^2^) to explore potential associations between anthropometric measures of obesity, body fat distribution, whole-body metabolism, and cerebral water content mapping. The research subjects underwent qMRI acquisition at 3 Tesla [21].

Interestingly, there were no global changes in water content associated with obesity. However, participants with higher BMI had increased water content values in the cerebellum, limbic lobe, and sub-lobular region. In addition, the dorsal striatum, hypothalamus, thalamus, fornix, anterior limb of the internal capsule, and posterior thalamic radiation showed the most substantial relationship with BMI. In a subgroup with available measurements for 50 patients, researchers further identified visceral adipose tissue as the most vital link between higher water content values and obesity. Additionally, the subjects with metabolic syndrome had the highest water content values in the hypothalamus and the fornix [21].

Accumulating evidence suggests that hypothalamus inflammation links to obesity-associated insulin resistance within that area. Nonetheless, whether brain inflammation is a cause or consequence of POCD in obese patients warrants full-scale investigation. By applying qMRI, researchers can further investigate and better understand the link between obesity and POCD and its underlying mechanism [21].

It is paramount to comprehend the relationship between POCD and obesity, including the pathophysiological mechanisms behind the association of obesity with POCD (see Table 2). By identifying possible therapeutic targets, medical practitioners can efficiently counsel obese patients before surgical procedures, monitor for signs of cognitive decline during recovery, and devise other strategies to stave off POCD or decrease cognitive deterioration among such patient cohorts post-surgery. By taking these steps, healthcare providers can help improve outcomes for their surgical patients by reducing the risk of developing this debilitating condition following surgery.

**Table 2:** Summary of the reviewed studies exploring the association between obesity and postoperative cognitive dysfunction (POCD) and potential mechanisms connecting the two conditions. The studies mentioned in this table met this systematic review’s inclusion criteria. Note: Kadoi and Goto (2006) [11] did not report the exact criteria they used to determine POCD from the neurocognitive test scores. Hence, it was unclear how they defined POCD. APOE-ε-4:apolipoprotein E-epsilon-4; BMI: body mass index.

Authors	Type of study	Level of clinical evidence	Sample size	Type of surgery	Cognitive assessment tools	Follow-up	POCD definition	Obesity parameters (BMI, body weight, risk factors for obesity)	Obesity association with POCD
Newman et al. (2001) [10]	Prospective, longitudinal	I	261	Coronary artery bypass grafting (CABG)	Digit Symbol subtest, Benton Visual Retention subtest, Randt Short-Story Memory Test, Digit Span subtest, and Trail Making Test	Before the surgery (baseline), before discharge, six weeks, six months, and five years after the surgery	≥1 standard deviation (SD) decline in the scores on tests of any one of the four domains of cognitive function	BMI or body weight not reported. Risk factors for obesity were assessed, including hypertension and diabetes	A significant number of patients had POCD post-surgery at follow-up assessments. Of those who suffered from POCD, a significant number of patients had risk factors for obesity. Of the POCD patients, 51% had hypertension and 14% had diabetes
Kadoi and Goto (2006) [11]	Prospective	II	88	CABG	Mini-Mental State Examination (MMSE), Rey Learning Test, Trail making A, Trail making B, Digit Span Forward, and Grooved Pegboard	Before the surgery (baseline) and six months after the surgery	Unclear	BMI or body weight not reported. In addition, risk factors for obesity were assessed, including hypertension, diabetes, and smoking	A significant number of patients had POCD post-surgery at the follow-up assessment. Logistic regression analysis showed risk factors for obesity, including hypertension, diabetes, smoking, and renal failure contributed to POCD
Heyer et al. (2005) [12]	Prospective	II	75	Carotid endarterectomy (CEA)	Digit Symbol subtest, Benton Visual Retention subtest, Randt Short-Story Memory Test, Digit Span subtest, and Trail Making Test	Before the surgery (baseline) and one month after the surgery	Summed total deficit score of ≥7 in any of the four cognitive domains	Obese: BMI > 30 kilograms (kg) per meter square (m^2^). Non-obese: BMI < 30 kg/m^2^	A significant number of patients had POCD post-surgery at the follow-up assessment. Obese patients and those who were APOE-ε-4-positive developed POCD after CEA significantly more than their counterparts
Zhu et al. (2014) [13]	Prospective	I	205	Total hip replacement surgery	MMSE	Before the surgery (baseline) and seven days after the surgery	≥1 SD decline on MMSE score	Obese: body weight ≥ 60 kg. Non-obese: body weight < 60 kg	A significant number of patients had POCD post-surgery at the follow-up assessment. Patients with POCD had a body weight of 61.1 ± 11 kg at baseline than their counterparts. However, it was not statistically significant
Pérez-Belmonte et al. (2015) [14]	Prospective	II	36	Off-pump CABG	Trail Making Test, Stroop Test, Selective Reminding Test, Verbal Fluency Tests (semantic and phonological), and Judgment of Line Orientation Test	Before the surgery (baseline) and after surgery at one, six, and 12 months	Percentile ranks of scores ≤18% on ≥1 neurocognitive test	Obese: BMI > 30 kg/m^2^. Non-obese: BMI < 30 kg/m^2^	A significant number of patients had POCD post-surgery at follow-up assessments. Furthermore, a significant subsection of the patients who developed POCD had obesity
Heyer et al. (2015) [15]	Prospective	I	585	CEA	Controlled Oral Word Association Test, Hopkins Verbal Learning Test, Buschke Selective Reminding Test, Rey-Osterrieth Complex Figure Copy and Recall Test, Grooved Pegboard, Finger Tapping Test, and Halstead-Reitan Trials A and B	Before the surgery (baseline) and within 24 hours after surgery	≥1.5 SD worse performance in all four domains of cognitive function and/or ≤2 SD worse performance in two or more domains of cognitive function	Obese: BMI > 30 kg/m^2^. Non-obese: BMI < 30 kg/m^2^	A significant number of patients had POCD post-surgery at follow-up assessments. Moreover, a significant number of the patients who developed POCD had obesity
He et al. (2019) [16]	Prospective	II	124	Gastrointestinal surgery (exact procedure unspecified)	Verbal learning and fluency test, Visuospatial Memory and Delayed Recall Test, Benton Judgment of Line Orientation, Trail Making Test Parts A and B, and Digit Span and Digit Symbol Substitution Test	Before the surgery (baseline) and seven days after the surgery	≥1.5 SD decline on two or more neuropsychological tests	Obese: body weight ≥ 60 kg. Non-obese: body weight < 60 kg	A significant number of patients had POCD post-surgery at the follow-up assessment. Patients with POCD had a body weight of 60.9 ± 9.8 kg at baseline than their counterparts. However, it was not statistically significant
Alla (2021) [17]	Prospective	II	84	Cholecystectomy	MMSE	Before the surgery (baseline) and 12 hours, and three and five days after the surgery	≥10% decline in MMSE score	Grade I obese: 30-34.9 kg/m^2^. Grade II obese: 35-39.9 kg/m^2^	At follow-up assessments, patients in the control group (who did not receive endothelioprotectors and anti-inflammatory agents) showed significantly more POCD after surgery than those in the two treatment groups (who received endothelioprotectors and anti-inflammatory agents, respectively)

## Conclusions

POCD can have profound effects if not promptly identified by medical professionals after surgery. Interestingly, there is a correlation between POCD and obesity. Therefore, following the PRISMA guidelines, this systematic review aimed to explore the link between the two. Based on clinical evidence, obesity is a substantial factor in the development of POCD, particularly in patients who are obese or at risk for obesity and undergo major surgical procedures like CABG, gastrointestinal procedures, or CEA. Furthermore, factors like APOE-ε-4 expression, changes in vascular endothelium integrity, and obesity-induced systemic inflammation significantly contribute to the development of POCD in obese patients following major surgeries. However, more research is required to investigate further the pathophysiological processes underlying the link between obesity and POCD. Therefore, future research endeavors should identify potential mechanisms leading to POCD in obese patients after major surgical procedures.
